# The Biological and Therapeutic and the Clinical Value of Radium and Roentgen Rays

**Published:** 1919-04

**Authors:** Henry Schmitz

**Affiliations:** Chicago, Illinois


### The Biological and Therapeutic Action and the Clinical Value of Radium and Roentgen Rays.

HENRY SCHMITZ, A. M., M. D., F. A. C. S.,

Chicago, Illinois.

From April 1, 1914, to October 1, 1918, we treated 283 patients with either the radium or the Roentgen ray or with a combination of these two or a combination of radio-therapy and surgery. The cases were clinically divided as follows:

Uterine carcinomata: 148, of which 21 were operable, 87 inoperable and 40 recurrent cancers. Rectal carcinomata: 43, of which 5 were operable and 38 inoperable and recurrent. Genito-urinary carcinomata: 32, of which 1 was operable and 31 inoperable and recurrent. Mammary carcinomata: 60, of which 13 were operable, 11 inoperable and 36 recurrent cancers. To these may be added 25 cases of bleeding myomata uteri; 50 cases of hemorrhagic myopathies; 8 cases of inflammatory myopathies; 1 of leukoplakia vulva and 1 of kraurosis vulva—a total of 85—making a grand total of 368 cases.

It is immediately seen that most of these diseases are usually surgically treated. If we decided to apply radium or Roentgen rays we did so because either surgery could not benefit the patient any more, while the rays gave some hope of palliation, or the rays would increase the efficacy of surgical treatment, or actinotherapy proved to be superior to surgical methods of treatment.

To enable us to decide whether a patient should be subjected to radium or the Roentgen treatment or a combination of these with surgery or a surgical method only, we should endeavor to answer these questions:

1. What can surgery accomplish in the treatment of disease?
2. What therapeutic value have radium and Roentgen rays in the treatment of disease?
3. Are there any differences in the action of radium and Roentgen rays?

An anatomical cure of a malignant disease can only result from a complete eradication of all tumor elements from the body of the bearer. If the malignancy is confined or localized to an area which can be completely and safely removed by a surgical procedure, it must be thus treated. The malignant disease is said to be operable. If the patient survives a time-period of five years following the surgical treatment, and during this time has been free of any recurrence of metastasis, he is said to be cured. It is, unfortunately, only very rarely that we observe cancer in such a localized, well defined state, that is, in its incipiency.

If the malignant growth has invaded the surrounding or distant tissues and organs to such an extent that we cannot remove the disease with the knife and with safety to the patient, a cure by surgical measures is absolutely precluded. Contrary we cause an auto-dissemination of malignant cells which rapidly leads to either a carcinomatosis or we irritate the tumor and cause it to grow with a boundless velocity. The patient has not been benefited by such therapy but has been rendered much more miserable than he would have been if left alone. The cancer has become inoperable and therefore hopeless. The absolute operability of a cancer can be determined with difficulty only. In about nine out of ten cases the patient comes to the surgeon at a time when the disease is beyond his reach. These facts explain the discouraging results of surgery in almost all forms of malignant disease in the human race.

In answering the second question we must consider the biological action of the rays on normal and abnormal tissue and cells. This action depends on the technique used in the administration of the rays and the histological character of the cell in which they become absorbed.

The factors observed in the use of radium are (1) the amount and compactness of the radium element used; (2) the filtration employed; (3) the distance between the source of the radiation and the tumor; (4) the duration of the application; and (5) the time interval between subsequent applications. In the therapy of the Roentgen ray we must observe (1) the source and the amount of the secondary current; (2) the size and kind of tube; (3) the hardness of tube; (4) distance of the anode from the tumor; (5) the time-period of duration of the application; and (6) the interval observed from one course of treatment to the next.

The effect of rays on living cells is both degenerative and destructive. The nearer the cells approach or remain in an undifferentiated, embryonal state, the more readily they undergo cytolysis or destruction. It does not matter whether the cells are normal or abnormal. This difference in the receptivity or sensitiveness of cells to the rays depends (1) on their age, both the momentary phase of the developmental period in which they happen to exist as well as the age of the host to which they belong, and (2) on the histologic species and the varieties in each one of these.

Cells which are in, or have not advanced far beyond, the embryonal and undifferential state as the basal cells of the skin and hair follicles, lymphoid cells, sex cells, as Graafin follicles and spermatozoa, are destroyed by a dosage of rays which would excite only a simple ruction in the surrounding mature tissues. The tissues of a child are much more easily altered by the rays than the corresponding structures in an adult. Tumors composed of embryonal cells succumb to the action of the rays sooner than the surrounding adult tissue elements. The difference in receptivity is acknowledged to be about as seven or six to one. The more undeveloped the embryonal cells of a malignant tumor remain, the more rapidly will the cells proliferate and the more receptive will they be to the action of the rays from radio-active substances.

An impediment in the growth of the tumor occurs soon after the beginning of the treatment. It is due (1) to a serious infiltration occurring in the area exposed to the rays, an enlargement of the cell nucleus causing an increase in the size of each individual cell and an obliteration of the capillaries due to an increase in the size of the endothelial cells; (2) to a degeneration of all the cell nucleus evidenced by cessation of mitosis, pyknosis, cytolysis and achromatism. These changes are of a traumatic nature and call forth an inflammatory reaction resulting in a lymphocyte and leukocyte infiltration, and a proliferation of the stroma. The latter is expressed by an enormous formation of young fibroblasts, which gradually develop into highly differentiated connective tissue cells and fibers. An excavation of portions of the tumor results. The spaces thus formed are filled by granulation and connective tissues which become covered by epithelium if degeneration occurred, or by scar tissue if necrosis took place.

The round cell infiltration and fibroblastic proliferation have still another significance. Their phagocytic faculties are instrumental in removing the debris of the necrobiotic cell elements. We do not wish to advance the impression that a microscopic examination of scar tissues would show a total absence of all abnormal cell elements, on the contrary, abnormal cells are always demonstrable on microscopic examination, although they are in a state of degeneration shown by absence of mitosis and every known variety of karyolysis, cytolysis and achromatism. It is impossible to state whether these abnormal cells are dormant or whether they are absolutely harmless and dead. Prime,* in a recent publication, considers that these processes effect the cell nucleus in preventing further mitosis. Such radiumized tissues will not grow when inoculated in mice. Radium does not kill the cells outright, but injures the nucleus in such a manner as to prevent further division which must eventually result in the death of the cell if its energy is expended in growth and division and not in a purely mechanical function.

The histologic changes in other tumors or tissues are identically the same, namely, impedient in the proliferation of the pathologic cell element and its subsequent death, round cell and connective tissue infiltration and phagocytosis, and finally heavy cicatrix formation, which is poor in blood supply and often resulting in a hyaline degeneration and softening, or connective tissue regeneration. The latter becomes covered with epithelium richly supplied with capillaries, while the former remains without an epithelial surface covering, but possesses a heavy, grayish pseudomembrane.

The technique of roentgenotherapy has been developed to a very exact state, that of radium is in its infancy, therefore by no means as yet perfect. We are still at variance whether the radium salts or its emanation should be used, whether large or small amounts should be employed, whether the duration of the application should or should not avoid any visible injury to the tissue such as burns and necrosis, whether we should filter out the beta-rays or use both beta and gamma rays. Personally, I incline to the view that we must treat the disease and not the patient. The surgeon is most successful in the treatment of malignant diseases who is most radical, who dares to work far out into the healthy tissues. Consequently the further reaching a degeneration or destruction of tissue is brought about by the rays, the better the remote results of radiotherapy must be.

Lastly we must determine when to use radium rays and when Roentgen rays. The therapeutic action of radium is confined to a radius of four centimeters, hence is purely local. That of the Roentgen rays emanating from a correctly adjusted Coolidge tube is far more intense and much more diffuse. The thera-

*Prime, F.: Observations on the Effects of Radium on Tissue Growth in Vitro, Journal of Cancer Research, April, 1917, 2.

peutic intensity of both can be multiplied by the crossfire method using as many portals of entrance as practicable in a given region of the body. Painstaking thoroughness and an unerring system bespeak results. We use radium in the body cavities applying it directly into or against the tumor mass and in surface carcinomata when purely local action is desirable. In all other instances we prefer the Roentgen ray.

The conclusion drawn from these statements may be briefly summarized as follows: Surgery removes diseased tissues and organs but cannot modify their pathological state. The gamma rays of radium element and the filtrated Roentgen rays can modify abnormal cells but cannot remove them. Therefore it is evident that a combination of these two methods of treatment would increase the efficiency of the one or other in many instances.

These are the viewpoints which we at present observe in the treatment of malignant or potentially malignant diseases. First, we ray with the combined gamma rays of radium and the hard filtered Roentgen rays to degenerate the abnormal cells; secondly, we eradicate the diseased tissues and surrounding areas surgically if one can do so and by measures as radical as possible, and finally we again apply the combined radium and Roentgen rays as thoroughly as permissible to destroy any diseased cell nests that inadvertently may have been left behind after the surgical eradication.

Prior to April, 1914, we only used the Roentgen rays either as a prophylactic following radical excision of disease, or as a palliative in inoperable cases. The results were very discouraging, excepting selected cases of myomata uteri, hemorrhagic myopathies and a few mammary carcinomata.

From April 1, 1914, to about April 1, 1915, we used radium rays either as a prophylactic after operations or as a palliative in inoperable disease. The primary results were very encouraging. We observed about 50 per cent of local healing in inoperable carcinomata. However, recurrences were the infallible rule. These occurred mostly in the parametrii and regional lymph glands.

Since April, 1915, we, therefore, used the combined treatment of radium and Roentgen rays, the former attacking the tumor locally and the latter acting on the surrounding tissues and organs and regional lymph glands.

We observed many cases of inoperable malignancy which were rendered entirely free of any palpatory abnormal findings.

As early as April, 1914, we decided to subject quite a number of these cases to radical excision by surgical methods. We used the electric cautery forceps of Downe either vaginally or abdominally, and then followed the operation by radiotherapy. We had many brilliant results. Finally we made it a rule to subject all cases of malignancy, whether operable, inoperable or border-line cases, to a preliminary treatment with radium and Roentgen rays, followed by an operation if the local and constitutional condition warranted the procedure and then again applied radium and Roentgen rays to preclude recurrence if possible. We are convinced that this plan forms the most ideal and advanced procedure of treatment in suitable cases.

The gross clinical results obtained in the more important of these diseases are as follows:

UTERINE CARCINOMATA.

| | Living. | Died.* | Not Heard From. | Total No. |
|---|---|---|---|---|
| Operable uterine carcinomata with pre- and post-operative raying. | 36-38-19 months. | 12-3 months. | 1 | 6 |
| Operable carcinomata with post-operative raying only. | 44-43-8-7-2 months. | 4-19-3-24-15-7-1 months. | 3 | 15 |

*Average duration of life in those who died is 11 months.

Of cases treated both prior to and after operation 50 per cent are living.

Of cases treated only after operation 33 per cent are living.

The patients living have been recently examined and are free from any recurrence.

| | Living. | Died.* | Not Heard From. | Total No. |
|---|---|---|---|---|
| Inoperable uterine carcinomata with rays and operation. | 19 months with recurrence.<br>15 months free of recurrence. | 9-3-31-5-5-8-7-11 months. | 2 died from septic peritonitis following operation. | 13 |

*Average duration of life in those who died is about 10 months.

| | Living. | Died.* | Not Heard From. | Total No. |
|---|---|---|---|---|
| Inoperable uterine carcinomata treated with rays only. | 1— 3 months.<br>1— 4 months.<br>5— 6 months.<br>1— 8 months.<br>2—11 months.<br>1—12 months.<br>1—13 months.<br>1—16 months.<br>2—17 months.<br>1—21 months.<br>1—30 months. | 2 after 1 month.<br>6 after 4 months.<br>3 after 3 months.<br>2 after 4 months.<br>1 after 5 months.<br>1 after 6 months.<br>1 after 8 months.<br>3 after 9 months.<br>3 after 10 months.<br>1 after 11 months.<br>2 after 12 months.<br>1 after 16 months.<br>1 after 21 months.<br>2 after 24 months.<br>1 after 26 months.<br>1 after 36 months. | 15 did not report.<br>6 died from effects of treatment.<br>5 too early to report. | |
| | 17 | 31* | 26 | 74 |

*The average duration of life was 9+ months.

| Recurrent uterine carcinomata. | Living. | Died.* | Not Heard From. | Total No. |
|---|---|---|---|---|
| | 24-24-15-4-4-4-2 months.<br>7 | 10-5-6-9-6-9-15-5-8-3-15-19-15-6-6-6-4-9-6 months.<br>19 | 9 did not report.<br>5 died from oper. procedure chiefly sepsis.<br>14 | 40 |

*Average duration of life was 8 1-3 months.

RECTAL CARCINOMATA.

| Operable | | Inoperable. | | Not Heard From. | Total No. |
|---|---|---|---|---|---|
| Living. | Died.* | Living | Died† | | |
| 33-13<br>2 | 12-26-16<br>3 | 24-22-18-15-13-8-6-4-4-4-4-3-2-2<br>14 | 6-3-17-6-30-6-6-2-6-9-8-6-3-7-4<br>15 | 8<br>1 died after colostomy.<br>9 | 43 |

Percentage living after one year 20 per cent.
*Duration of life of those operable, 18 months.
†Duration of life of those inoperable, 7½ months.

GENITO-URINARY CARCINOMATA.

| Operable. | | Inoperable. | | Not Heard From. | Total No. |
|---|---|---|---|---|---|
| Living. | Died. | Living. | Died. | | |
| | 3<br>1 | 16-36-27-18-9-7-7-6-8-7-4-4-5-3-5-2<br>16 | 5-2-6-6-8-6-2<br>7 | 4<br>2 died from urethral fever and subsequent congestion of kidneys.<br>1 died from advanced carcinoma soon after beginning of treatment.<br>1 died from sepsis after cautery.<br>8 | 32 |

MAMMARY CARCINOMATA.

| Operalbe | | | Recurrent | | | Inoperable | | |
|---|---|---|---|---|---|---|---|---|
| Living. | Died. | No Report. | Living. | Died. | No Report. | Living. | Died. | No Report. |
| 32-22-20-19-15-13-7-5-4-4-5<br>11 | 1<br>1 | 1<br>1 | 34-21-17-17-15-13-13-9-12-9-4-11<br>12 | 46-9-3-9-15-8-½-6-24-28-3-4-16-17-2-3-11-9-9-1<br>20 | 4<br>4 | 21-7-5-3<br>4 | 6-2<br>2 | 1 shock.<br>4<br>5 |

Operable mammary cancers living 11; died or no report 2; i. e., 85 per cent living.

Recurrent mammary cancers living 12; died or no report 24, i. e., 33+ per cent living.

Inoperable mammary cancers living 4; died or no report 7; i. e., 36 per cent living.

The results obtained in the treatment of carcinomata are of course without any statistical value, as the time-honored period of five years has not yet elapsed in a single instance. If we compare the results with purely surgical statistics as given by Reuben Peterson, John G. Clark, Howard Kelly, Kronig, James F. Percy and Mayo amongst others, we may be permitted to regard the results as very encouraging and promising. They may not be as brilliant as those obtained by Kelly and Burnam in uterine carcinomata, or Janeway in cancers of the jaw, yet they compare favorably with those reported by John G. Clark, the London and Manchester Radium Institutes and the Harvard University Cancer Commission. A perusal of our results in mammary cancers proves the obvious value of radiotherapy in operable as well as recurrent and inoperable cancers. The fact that the results in mammary cancers are better than in pelvic malignancy may be explained by the greater technical difficulties in diagnosis and surgical as well as ray treatment in the latter.

Eighty-five cases of benign diseases of the female genital organs were treated by actinotherapy. Fifty had a hemorrhage and eight an inflammatory myopathy; twenty-five myomata uteri, one a leukoplakia and one a kraurosis of the vulva. The indications for the ray treatment are the presence of an essential menorrhagia or an essential leucorrhea in the myopathies and uterine hemorrhages in the myomata which do not yield to medicinal treatment. Submucous pedunculated, cervical and degenerating myomata also, form a contradindication. The possibility of the presence of malignancy must be absolutely excluded before treatment is instituted. If in doubt radical excision must be practiced. Procrastination would be criminal. Amongst the hemorrhagic myopathies we had one recurrence, amongst the inflammatory myopathies three failures, and amongst the myomata two negative results. The leukoplakia vulvae yielded to the rays, while the kraurosis did not even improve.

In conclusion we feel justified to formulate the following principles:

1. Operable carcinomata should receive a preliminary application of radium and Roentgen rays to cause a degeneration of the abnormal cells. Subsequent radical extirpation which preferably should be done with cautery knives and clamps must be followed by another course of radium or Roentgen therapy or both, to insure as intense a degeneration of the disease area as possible.

2. Radium and Roentgen rays form the ideal palliative treatment in inoperable and recurrent malignant diseases.

Cures are very few. The advantages as a palliative are, however, superior to any other known method. Whether surgical eradication should be recommended after a local cure has been obtained by radiotherapy cannot as yet be stated. Microscopic examination of radiumized tissue seems to justify such a procedure.

3. In the benign hemorrhagic diseases of the uterus and the inflammatory myopathies radium has proven of the utmost therapeutic value. Without causing mutilation it brings about a cessation of the hemorrhages and leucorrhea and very frequently a disappearance of the myoma.

